# Supplementary material for: A colorimetric method for detecting virulent bacteriophage to Vibrio cholerae in fecal and environmental samples
Source: PLoS Negl Trop Dis. 2025 Dec 18;19(12):e0013674. doi: 10.1371/journal.pntd.0013674 (PMC12725621; doi:10.1371/journal.pntd.0013674)
Supplement: S1 File — (DOCX) [file pntd.0013674.s001.docx]

**Supplementary Information**

**Table A.** These data are from Figure 2 which used a one-step method. The phages were serially diluted in PBS and spiked into Luria-Bertani broth. The columns show the concentration of the phages, the sample type, the time of amplification, and the OD_600_ ratio (sample OD / negative control OD).

| **Sample type** | **ICP1 dose (PFU)/ML** | **Sample type** | **time of amplification(hour)** | **Ratio-OD600** |
| --- | --- | --- | --- | --- |
| LB broth | 0 | dye only, no bacteria (dye control) | 2 | 14.5 |
|  | 0 | dye + bacteria, no phage (negative control) | 2 | 1 |
|  | 4 | dye+bacteria+phage | 2 | 3.9 |
|  | 40 | dye+bacteria+phage | 2 | 7.4 |
|  | 400 | dye+bacteria+phage | 2 | 10.4 |
|  | 0 | dye only, no bacteria (dye control) | 2.5 | 12 |
|  | 0 | dye + bacteria, no phage (negative control) | 2.5 | 1 |
|  | 4 | dye+bacteria+phage | 2.5 | 4.7 |
|  | 40 | dye+bacteria+phage | 2.5 | 8.6 |
|  | 400 | dye+bacteria+phage | 2.5 | 9.8 |
|  | 0 | dye only, no bacteria (dye control) | 3 | 11.8 |
|  | 0 | dye + bacteria, no phage (negative control) | 3 | 1 |
|  | 4 | dye+bacteria+phage | 3 | 9 |
|  | 40 | dye+bacteria+phage | 3 | 9.4 |
|  | 400 | dye+bacteria+phage | 3 | 9.9 |

**Table B**. These data are from Figure 3a which used a one-step method with bile peptone, stool and bay-water using filtration to generate bacteria-free samples. The columns show the concentration of the phages, the sample type, the time of amplification, and the OD_600_ ratio (sample OD / negative control OD).

| **Sample type** | **ICP1 dose PFU/ML** | **Sample type** | **Ratio-OD600** |
| --- | --- | --- | --- |
| Bile peptone broth | 0 | dye only, no bacteria (dye control) | 11.7 |
|  | 0 | Dye + bacteria, no phage (negative control) | 1 |
|  | 4 | Dye+bacteria+phage | 1 |
|  | 40 | Dye+bacteria+phage | 8.9 |
|  | 400 | Dye+bacteria+phage | 7.3 |
| Stool | 0 | dye only, no bacteria (dye control) | 8 |
|  | 0 | Dye + bacteria, no phage (negative control) | 1 |
|  | 4 | Dye+bacteria+phage | 2 |
|  | 40 | Dye+bacteria+phage | 3.1 |
|  | 400 | Dye+bacteria+phage | 7.2 |
| Bay water | 0 | dye only, no bacteria (dye control) | 12.4 |
|  | 0 | Dye + bacteria, no phage (negative control) | 1 |
|  | 4 | Dye+bacteria+phage | 1 |
|  | 40 | Dye+bacteria+phage | 5.6 |
|  | 400 | Dye+bacteria+phage | 9.2 |

**Table C**. These data are from Figure 3b which used a one-step method with bile peptone, stool and bay-water with chloroform to lyse and generate bacteria-free samples. The columns show the concentration of the phages, the sample type, the time of amplification, and the OD_600_ ratio (sample OD / negative control OD).OD).

| **Sample type** | **ICP1 dose** | **Sample type** | **Ratio-OD600** |
| --- | --- | --- | --- |
| Bile peptone broth | 0 | dye only, no bacteria (dye control) | 13.1 |
|  | 0 | Dye + bacteria, no phage (negative control) | 1 |
|  | 4 | Dye+bacteria+phage | 0.7 |
|  | 40 | Dye+bacteria+phage | 0.9 |
|  | 400 | Dye+bacteria+phage | 7.5 |
| Stool | 0 | dye only, no bacteria (dye control) | 5.7 |
|  | 0 | Dye + bacteria, no phage (negative control) | 1 |
|  | 4 | Dye+bacteria+phage | 1.8 |
|  | 40 | Dye+bacteria+phage | 2 |
|  | 400 | Dye+bacteria+phage | 2.3 |
| Bay water | 0 | dye only, no bacteria (dye control) | 17.8 |
|  | 0 | Dye + bacteria, no phage (negative control) | 1 |
|  | 4 | Dye+bacteria+phage | 1.2 |
|  | 40 | Dye+bacteria+phage | 1 |
|  | 400 | Dye+bacteria+phage | 2.5 |

**Table D**. These data are from Figure 4 which used the two-step procedure with bile peptone, stool and bay-water using chloroform to lyse and generate bacteria-free samples. The columns show the concentration of the phages, the sample type, the time of amplification, and the OD_600_ ratio (sample OD / negative control OD).

| **Sample type** | **ICP1 dose** | **Sample type** | **Ratio-OD600** |
| --- | --- | --- | --- |
| Bile peptone broth | 0 | dye only, no bacteria (dye control) | 12 |
|  | 0 | Dye + bacteria, no phage (negative control) | 1 |
|  | 4 | Dye+bacteria+phage | 2.3 |
|  | 40 | Dye+bacteria+phage | 1.5 |
|  | 400 | Dye+bacteria+phage | 8 |
| Stool | 0 | dye only, no bacteria (dye control) | 4.2 |
|  | 0 | Dye + bacteria, no phage (negative control) | 1 |
|  | 4 | Dye+bacteria+phage | 1.2 |
|  | 40 | Dye+bacteria+phage | 2 |
|  | 400 | Dye+bacteria+phage | 2.3 |
| Bay water | 0 | dye only, no bacteria (dye control) | 8.9 |
|  | 0 | Dye + bacteria, no phage (negative control) | 1 |
|  | 4 | Dye+bacteria+phage | 3.8 |
|  | 40 | Dye+bacteria+phage | 5.8 |
|  | 400 | Dye+bacteria+phage | 5.8 |
